# Does the Glucocorticoid Stress Response Make Toads More Toxic? An Experimental Study on the Regulation of Bufadienolide Toxin Synthesis

**DOI:** 10.1093/iob/obad021

**Published:** 2023-06-05

**Authors:** B Üveges, C Kalina, K Szabó, Á M Móricz, D Holly, C R Gabor, A Hettyey, V Bókony

**Affiliations:** Department of Evolutionary Ecology, Plant Protection Institute, Centre for Agricultural Research, Eötvös Loránd Research Network, Herman Ottó út 15, 1022 Budapest, Hungary; Molecular Ecology and Evolution at Bangor, School of Natural Sciences, Bangor University, Environment Centre Wales, Bangor LL57 2UW, UK; Department of Evolutionary Ecology, Plant Protection Institute, Centre for Agricultural Research, Eötvös Loránd Research Network, Herman Ottó út 15, 1022 Budapest, Hungary; Department of Ecology, Institute of Biology, University of Veterinary Medicine, István u. 2, 1078 Budapest, Hungary; Division of Clinical Immunology, Department for Internal Medicine, Faculty of Medicine, University of Debrecen, Móricz Zsigmond út 22, 4032 Debrecen, Hungary; Department of Pathophysiology, Plant Protection Institute, Centre for Agricultural Research, Eötvös Loránd Research Network, Herman Ottó út 15, 1022 Budapest, Hungary; Department of Evolutionary Ecology, Plant Protection Institute, Centre for Agricultural Research, Eötvös Loránd Research Network, Herman Ottó út 15, 1022 Budapest, Hungary; Department of Evolutionary Ecology, Plant Protection Institute, Centre for Agricultural Research, Eötvös Loránd Research Network, Herman Ottó út 15, 1022 Budapest, Hungary; Department of Biology, College of Science and Engineering, Texas State University, 601 University Dr., San Marcos, TX 78666, USA; Department of Evolutionary Ecology, Plant Protection Institute, Centre for Agricultural Research, Eötvös Loránd Research Network, Herman Ottó út 15, 1022 Budapest, Hungary; Department of Evolutionary Ecology, Plant Protection Institute, Centre for Agricultural Research, Eötvös Loránd Research Network, Herman Ottó út 15, 1022 Budapest, Hungary; Department of Ecology, Institute of Biology, University of Veterinary Medicine, István u. 2, 1078 Budapest, Hungary

## Abstract

Chemical defense is a crucial component of fitness in many organisms, yet the physiological regulation of defensive toxin synthesis is poorly understood, especially in vertebrates. Bufadienolides, the main defensive compounds of toads, are toxic to many predators and other natural enemies, and their synthesis can be upregulated by stressors, including predation risk, high conspecific density, and pollutants. Thus, higher toxin content may be the consequence of a general endocrine stress response in toads. Therefore, we hypothesized that bufadienolide synthesis may be stimulated by elevated levels of corticosterone (CORT), the main glucocorticoid hormone of amphibians, or by upstream regulators that stimulate CORT production. To test these alternatives, we treated common toad tadpoles with exogenous CORT (exoCORT) or metyrapone (MTP, a CORT-synthesis inhibitor that stimulates upstream regulators of CORT by negative feedback) in the presence or absence of predation cues for 2 or 6 days, and subsequently measured their CORT release rates and bufadienolide content. We found that CORT release rates were elevated by exoCORT, and to a lesser extent also by MTP, regardless of treatment length. Bufadienolide content was significantly decreased by treatment with exoCORT for 6 days but was unaffected by exposure to exoCORT for 2 days or to MTP for either 6 or 2 days. The presence or absence of predation cues affected neither CORT release rate nor bufadienolide content. Our results suggest that changes in bufadienolide synthesis in response to environmental challenges are not driven by CORT but may rather be regulated by upstream hormones of the stress response.

## Introduction

Using toxic or noxious chemical compounds for protection from natural enemies, such as predators, pathogens, parasites, and competitors, is widespread in the animal kingdom (Blum 1981; Pawlik 1993; Brodie 2009; Casewell et al. 2013). Chemical defense is important from the perspective of ecology and life-history evolution, as chemically protected animals can have a longer life span (Hossie et al. 2013) and occupy a larger niche space (Arbuckle et al. 2013). Toxins can also influence the survival not only of the defended animals themselves (Hayes et al. 2009; Llewelyn et al. 2012), but also of other species. For instance, when consuming unusually toxic prey, predators can suffer high mortality and population declines, as shown by the infamous example of invasive cane toads (*Rhinella marina*) that endanger native fauna (Shine 2010). On the other hand, toxins can also change trophic interactions and community structure if predators learn to avoid the consumption of toxic animals and switch to alternative prey (Webb et al. 2008; Nelson et al. 2010, 2011). Furthermore, the presence of effective toxins may also induce co-evolutionary arms races between prey that produce them and predators that adapt to their consumption by evolving toxin resistance (Brodie and Brodie 1990; Ujvari et al. 2015). Despite the widespread occurrence of chemical defense and its importance for multiple aspects of biology, the physiological regulation of toxin synthesis remains poorly known in a plethora of species.

Toads (Bufonidae, Amphibia) produce skin secretions containing, among others, bufadienolides as defensive compounds, which can be effective against predators and possibly also against pathogens (Toledo and Jared 1995; Filho et al. 2005; Tempone et al. 2008; Barnhart et al. 2017; Üveges et al. 2019). Bufadienolides are potent inhibitors of Na^+^/K^+^-ATPase (Pierre and Xie 2006; Schoner and Scheiner-Bobis 2007; Lingrel 2010) and can cause serious symptoms upon ingestion, including nausea, convulsions, hypertension, cardiac arrhythmia, and even death (Toledo and Jared 1995; Chen and Huang 2013; Kamboj et al. 2013). The rate of bufadienolide synthesis in toads can be increased by various environmental factors such as predation risk (Hettyey et al. 2019), high conspecific density (Bókony et al. 2018; Üveges et al. 2021), reduced food availability (Üveges et al. 2017), and anthropogenic pollutants (Bókony et al. 2017). This high diversity of inducing stressors raises the question whether upregulated bufadienolide synthesis is a consequence or part of the generalized endocrine stress response.

**Fig. 1:**
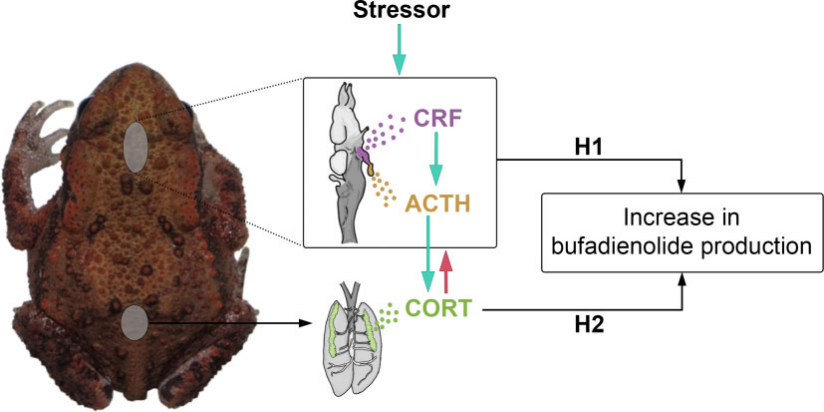
Schematic representation of the HPI axis and potential physiological regulatory pathways of bufadienolide synthesis in common toads. Green arrows indicate upregulation, whereas a red arrow indicates negative feedback. Abbreviations: CRF, corticotropin-releasing factor; ACTH, adrenocorticotropic hormone; CORT, corticosterone; H1, hypothesis 1; H2, hypothesis 2. Photo of a juvenile toad by Nikolett Ujhegyi, used with permission.

The endocrine stress response, which is highly conserved across vertebrates (Denver 2009; Godoy et al. 2018), serves to maintain homeostasis by allostasis and ensure survival by responding to environmental perturbance (McEwen 1998; McEwen and Wingfield 2003; Koolhaas et al. 2011). One integral part of this system is the hypothalamic–pituitary–adrenal (HPA, or hypothalamic–pituitary–interrenal [HPI]) axis (Fig. 1). The perception of environmental stressors triggers the discharge of corticotropin-releasing factor (CRF) from the hypothalamus, which in turn induces production of adrenocorticotropic hormone (ACTH) in the anterior pituitary gland. ACTH stimulates the adrenal glands (or interrenal glands in amphibians and fish; Denver 2009) to produce glucocorticoid hormones (GCs, notably cortisol and corticosterone [CORT]). GCs modulate a multitude of physiological processes, including metabolism and immune response, and facilitate recovery after stress by promoting energy uptake of cells and tissues via gluconeogenesis (Sterling and Eyer 1988; McEwen 1998, 2004; McEwen and Wingfield 2003; Denver 2009; Godoy et al. 2018). On the other hand, chronic stress might lead to long-term elevated levels of circulating GCs, resulting in adverse effects such as suppression of the immune system and cardiovascular, neurological, and metabolic disorders (Sterling and Eyer 1988; McEwen 1998, 2004; McEwen and Wingfield 2003; Denver 2009; Godoy et al. 2018). To keep GC levels in check, the HPA/HPI axis is under internal control by negative feedback loops. For instance, high levels of circulating GCs inhibit the release of CRF and ACTH, thus returning GC secretion to baseline levels (Gjerstad et al. 2018; Godoy et al. 2018; Herman et al. 2020).

Various environmental stressors that trigger the GC response, such as predation risk (Berger et al. 2007; Middlemis Maher et al. 2013), intraspecific competition (Glennemeier and Denver 2002a; Eraud et al. 2008; Braasch et al. 2014), and anthropogenic pollutants (Ye et al. 2015; Gabor et al. 2018; Monclús et al. 2018), also elevate the synthesis of bufadienolide toxins (Bókony et al. 2017, 2018; Üveges et al. 2017, 2021; Hettyey et al. 2019). Further similarities also suggest a physiological link between the two groups of compounds. Both CORT and bufadienolides are synthesized from the precursor cholesterol (Payne and Hales 2004; Fedorova et al. 2015), and the synthesis of cardiotonic steroids, the compound family bufadienolides belong to, has been suggested to be upregulated by ACTH in mammals (Vinge et al. 1993; Laredo et al. 1994, 1995; Li et al. 1994; Yamada et al. 1997; Sophocleous et al. 2003; Dostanic-Larson et al. 2005). However, the specific physiological regulatory mechanisms behind the synthesis of bufadienolides are still largely unknown (although see Fedorova et al. 1998; Dostanic-Larson et al. 2005), and explicit information about how GCs affect bufadienolide synthesis is, to our knowledge, entirely lacking.

**Fig. 2:**
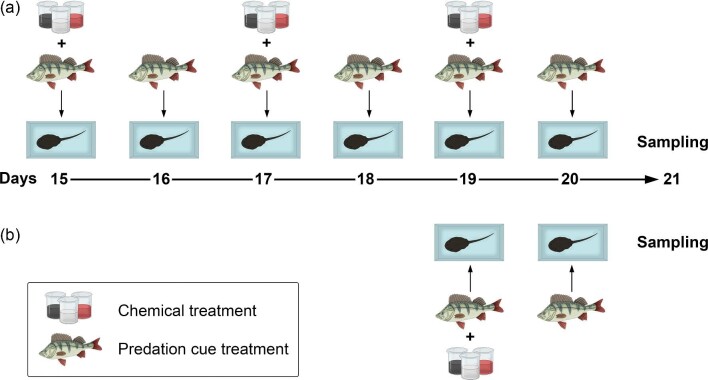
Experimental design applied to containers of tadpoles during the last week of the experiment (treatment period), beginning with day 15. (**a**) treatment length 6 days, (**b**) treatment length 2 days. Beakers represent changing the tadpoles’ rearing water with water containing exogenous chemicals (CORT, MTP) or the control solution. Perch represent the addition of reconstituted soft water (control tadpoles) or water from the fish's tank + tadpole homogenate. Icons of beakers used in the figure are licensed by Biorender.

Given the information presented above, we hypothesized that the HPI axis may provide a key to understanding the proximate mechanism of what makes toads more (or less) toxic. Specifically, we formulated two alternative hypotheses (Fig. 1). According to hypothesis 1 (H1), increased CORT levels upregulate bufadienolide synthesis directly. Conversely, hypothesis 2 (H2) postulates that bufadienolide production is regulated by upstream hormones of the HPI axis, which simultaneously increase the levels of both CORT and bufadienolides (Fig. 1). To test these alternatives, we reared tadpoles of the common toad (*Bufo bufo*) in the laboratory and exposed them to treatments with exogenous CORT (exoCORT), metyrapone (MTP, a CORT synthesis inhibitor), or a solvent control. We predicted that treatment with exoCORT would lead to increased bufadienolide content if H1 was true, but to decreased bufadienolide content if H2 was true (due to negative feedback loops inhibiting expression of upstream regulators; Fig. 1). MTP is used to test HPA functionality by blocking CORT synthesis, which in turn can stimulate the secretion of CRF and ACTH (Gjerstad et al. 2018; Godoy et al. 2018; Herman et al. 2020). Thus, in toad tadpoles treated with MTP, we expected to observe a decrease in bufadienolide content if H1 was true, but an increase if H2 was true (Fig. 1). Because earlier results indicated that MTP may not reduce CORT in the absence of stressors (Gabor et al. 2018), we combined the chemical treatments with a predator treatment (presence or absence of predation cues; Fig. 2) since predator presence is a natural stressor highly relevant to chemical defense. To discriminate between the acute and chronic effects of CORT on bufadienolide synthesis, we applied the treatments for either 2 or 6 days (Fig. 2).

## Materials and Methods

### Experimental procedures

We collected 50 common toad eggs from each of 8 clutches (egg strings) in April 2019 from a pond located in the Pilis Mountains, Hungary (Zánkói rét, 47.740515°N, 19.016276°E). We transported eggs to the laboratory at the Plant Protection Institute in Budapest. Eggs and embryos from each clutch were kept together in 1 L reconstituted soft water (RSW, 48 mg/L NaHCO_3_, 30 mg/L CaSO_4 _× 2H_2_O, 61 mg/L MgSO_4 _× 7H_2_O and 2 mg/L KCl dissolved in UV treated, reverse osmosis filtered, aerated tap water) in the laboratory until hatching, after which the water level was increased to 9 L (container size: 26.8 × 36.9 × 16.6 cm, width × length × height) until tadpoles reached the free-swimming stage (developmental stage 25, Gosner 1960). The air temperature in the laboratory was 20.1 ± 1.1°C, resulting in 19.0 ± 0.2°C water temperature in tadpole containers. The light:dark cycle was adjusted weekly to simulate natural changes in photoperiod.

We began the experiment when the tadpoles reached the free-swimming stage (day 1). We haphazardly selected 36 toad tadpoles from each clutch and placed them individually into 2-L plastic boxes (11.8 × 16.5 × 12.8 cm) containing 1 L RSW. We fed experimental tadpoles throughout the study twice a week ad libitum. To ensure that cholesterol, the precursor for both endogenous CORT and bufadienolides, was not a limiting factor for the tadpoles, we fed them with a 20:1 mixture of slightly boiled, chopped spinach and minced axolotl food (JBL NovoLotl M, fat content 12%). We changed the water of tadpoles twice a week for 2 weeks until the start of the treatment period (see below).

On day 15 of the experiment, we assigned the tadpoles randomly to treatments with exogenous chemicals combined with the simultaneous presence or absence of predation cues for either 6 or 2 days in a fully crossed design (see below for details, Fig. 2). We applied treatments during the third week of the experiment, when toxin production of common toad tadpoles reacts strongest to environmental change (Ujszegi et al. 2017; Üveges et al. 2017). We applied treatments only for relatively short periods because CORT inhibits the growth and development of tadpoles (Glennemeier and Denver 2002b), and we wanted to avoid inducing a strong effect on the formation of cells producing bufadienolides (Delfino et al. 1995). Experimental containers were arranged in a randomized block design with each block containing one individual from each treatment × clutch combination. We repeated each treatment × clutch combination three times (3 blocks). Therefore, the total number of experimental units was 288 toad tadpoles (8 clutches × 2 treatment lengths (6 or 2 days) × 3 chemical treatments (exoCORT, MTP, and control) × 2 predation cue treatments (cues present or absent) × 3 blocks). During the experiment, three tadpoles died for unknown reasons, reducing the final sample size to 285.

To manipulate the internal CORT levels of experimental tadpoles, we exposed them either to exoCORT or MTP (Sigma–Aldrich). As a solvent control treatment, the third group of the tadpoles received the same concentration of ethanol (EtOH) as their conspecifics in the exoCORT and MTP treatments. We did not use a control treatment with RSW only (without EtOH), because previous studies showed that the low concentration of ethanol used in our experiment does not affect the development and growth of tadpoles (Rohr et al. 2013; Young et al. 2020), including common toad larvae (Verebélyi 2017). Right before the start of treatments, we created stock solutions of CORT (cc. 25 mg/ml) and MTP (cc. 30 mg/ml) by dissolving them in 96% EtOH and stored these in the dark at 4°C until use. Similar to previous studies (Glennemeier and Denver 2002a, 2002b; Forsburg et al. 2019; Gabor et al. 2019), we diluted stock solutions to final concentrations of 125 nM exoCORT, 110 μM MTP, and 1 μL/L EtOH by adding them to RSW before each change of the rearing water of tadpoles. These concentrations of exoCORT and MTP increase and decrease, respectively, CORT in amphibian larvae, by approximately 50% (Glennemeier and Denver 2002a, 2002b; Forsburg et al. 2019; Gabor et al. 2019). We exposed tadpoles to exogenous chemicals by changing the water of experimental animals using these freshly prepared solutions on days 15, 17, and 19 (6-day treatment) or only on day 19 (2-day treatment, Fig. 2). This way, tadpoles were able to absorb exoCORT and MTP from their rearing water (Hayes and Wu 1995; Glennemeier and Denver 2002a, 2002b; Middlemis Maher et al. 2013; Gabor et al. 2018).

During the treatment period, half of the experimental tadpoles received cues indicating the presence of common perch (*Perca fluviatilis*), a predatory fish to which common toad larvae can respond with increased bufadienolide production (Hettyey et al. 2019). Predator cues were provided by six fish (body mass, mean ± SD: 39.28 ± 7.07 g) kept under laboratory conditions in 130 L aerated RSW, which was changed once a week. Fish were fed 3 times a week with 40 live agile frog (*Rana dalmatina*) tadpoles and Tubifex worms ad libitum during the first 2 weeks of the experiment. From day 14 until the termination of the experiment, we fed fish daily with 6.06 ± 0.38 g (mean ± SD) live agile frog tadpoles only. We fed fish with tadpoles to simulate a high risk of tadpole predation in general and to thereby elicit strong antipredator responses in toad larvae (Laurila et al. 1997; Schoeppner and Relyea 2005; Hettyey et al. 2015). We did not offer common toad tadpoles as food, because fishes often, but not always, avoid consuming them (Üveges et al. 2019), which would have made it difficult to obtain replicable quantities of chemical cues of predation risk. Agile frog tadpoles used for feeding fish were collected as eggs in a pond in the Pilis Mountains, Hungary (Jávor tó, 47.713646°N, 19.019953°E). Agile frog tadpoles and toad tadpoles used for the preparation of stimulus water (see below) were raised in the same way as focal tadpoles until developmental stage 25, after which they were reared in outdoor mesocosms. After the termination of the experiment, all remaining tadpoles were released to their pond of origin.

During the treatment period, we created stimulus water daily by homogenizing 276.33 ± 27.82 g (mean ± SD, days 15–18) and 487.55 ± 14.35 mg toad tadpoles (days 19–20) with a blender in ca. 50 mL RSW (Benard and Fordyce 2003; Hettyey et al. 2019). This resulted in the immediate death of tadpoles. We opted for this method, because we did not want chemicals (e.g., MS222) used for euthanizing tadpoles to affect our experimental animals (Achtymichuk et al. 2022). We then added this homogenate to 2 and 3.5 L water, respectively, taken from the fish tank 2–3 h after feeding the fish. This way, the concentration of chemical cues in the stimulus water was kept constant during the whole treatment period. We refilled the fish tank to the original volume with RSW after each stimulus-water preparation. We added the tadpole homogenate to ensure that experimental tadpoles were exposed to sufficiently high concentrations of conspecific prey-borne cues of predation to elicit strong antipredator responses (Laurila et al. 1997; Schoeppner and Relyea 2005; Hettyey et al. 2015). During the treatment period, we added 20 mL of stimulus water (predation cue treatment) or clean RSW (control treatment) to the water of focal tadpoles daily using a pipette. As a result, experimental tadpoles were exposed to chemical cues corresponding to 34.05 ± 1.15 mg/L fish (sources of kairomones, mean ± SD), 0.89 ± 0.01 mg/L agile frog tadpoles (chemical cues released by the fish digesting frog tadpoles and sources of alarm pheromones or “Schreckstoff,” von Frisch 1942), as well as to 2.66 ± 0.21 mg/L homogenized conspecifics (sources of chemical cues of conspecifics released by mechanical damage).

We terminated the experiment on day 21 after applying a noninvasive water-borne hormone sampling method that measures the amount of CORT released by tadpoles into the surrounding water (Gabor et al. 2013a; Baugh et al. 2018; Forsburg et al. 2019). This method provides a CORT measurement that is repeatable within individuals, correlates with plasma levels, and responds to ACTH challenge (Gabor et al. 2013a, 2016; Forsburg et al. 2019). All experimental tadpoles were transferred from their rearing containers to individual 0.5 L plastic cups containing 100 mL RSW in a random order between 09:00 and 10:00 am. To avoid transferring exogenous chemicals, we first poured the rearing water of experimental animals through a stretched-out aquarium net. Then, we used a dry piece of mosquito net (single-use for each animal) to transfer each tadpole one by one from the surface of the aquarium net into the plastic cups. After each tadpole spent 1 h in the cup, we poured the water into individual polypropylene (PP) containers and stored the water samples at –20°C until analysis. We then preserved each tadpole in a microcentrifuge tube containing HPLC (high-performance liquid chromatography) grade absolute methanol for chemical analysis of their toxin content, which resulted in the immediate death of tadpoles. The developmental stage of tadpoles (median: 35, range: 32–37, Gosner 1960) was determined by inspecting the preserved tadpoles under a stereomicroscope.

The study was approved by the Environment Protection and Nature Conservation Department of the Pest County Bureau of the Hungarian Government (PE-06/KTF/8060–1/2018, PE-06/KTF/8060–2/2018, PE-06/KTF/8060–3/2018, and PE/EA/295–7/2018,) as well as by the Ethics Committee of the Plant Protection Institute, Centre for Agricultural Research, Eötvös Loránd Research Network.

### Preparation of hormone samples and analysis of CORT release rates

We extracted the CORT content of water samples using C18 solid phase extraction (SPE) columns (Sep-Pak Vac, 3cc/500mg, Waters Inc.) following an established protocol (Gabor et al. 2013a, 2016; Forsburg et al. 2019). We primed each SPE column with 4 mL HPLC grade abs. methanol and 4 mL reverse-osmosis filtered water. Samples were forced through SPE columns using Tygone tubing under a vacuum. We eluted samples with 4 mL HPLC grade absolute methanol into 5 mL PP microcentrifuge tubes. The eluted solution was kept at –20°C until samples were transferred into 15 mL PP centrifuge tubes and dried out by vacuum centrifugation (40°C, 1000 rpm, 100 Pa) and frozen again. Samples were analyzed using a competitive enzyme-linked immunosorbent assay (ELISA, Cayman Chemical Inc.) following the manufacturers’ protocol and based on the previously published methodology (Gabor et al. 2013a, 2016). We dissolved each sample in 12.5 μL of 96% EtOH, vortexed them for 5 min, and subsequently added 237.5 μL ELISA buffer to obtain a final volume of 250 μL. Samples obtained this way were homogenized on an orbital shaker for 45 min to promote dissolution of CORT in the ethanol–buffer solution before transferring them to plates. Two samples were lost during the preparation process, leaving us with 283 samples to be analyzed for CORT content. The ELISA color reaction was quantified at 412 nm wavelength using a microplate reader (Synergy HT, Bio-Tek Instruments).

Because we had no prior information on the range of waterborne CORT concentration in common toad samples, we prepared a dilution series from six samples to estimate the approximate range of sample concentrations in our study. To account for variation in the CORT concentrations of our samples, we then analyzed two dilutions of each sample that would potentially fit in the linear region of the standard curve of the CORT standard. For subsequent statistical analyses, we used CORT concentrations of the dilution with the best fit. CORT concentrations were corrected for dilution. Due to limited ELISA kit availability, we could not analyze samples of the same dilution in duplicate. Therefore, the intra-assay coefficient of variation (CV) could not be calculated. The inter-assay CV, calculated from concentrations of the CORT standard, was 9.4%.

### Preparation of toxin samples and analysis of bufadienolide content

We prepared and analyzed bufadienolide samples following an established protocol (for a detailed description of methods, see the Supplementary material and Hettyey et al. 2019; Üveges et al. 2021). Briefly, we homogenized preserved tadpoles and dried the resulting homogenates, then we measured the dry mass of samples and re-dissolved them in 1 mL HPLC-grade absolute methanol. Finally, we filtered samples and analyzed the resulting solution using HPLC with diode-array detection and mass spectrometry (HPLC-DAD-MS). Bufadienolides were identified based on their characteristic peaks in the UV spectrum (Benard and Fordyce 2003; Hagman et al. 2009; Üveges et al. 2017, 2019; Bókony et al. 2018; Hettyey et al. 2019), by co-injection with selected bufadienolide standards and by additionally analyzing a bulk toxin sample from 49 juvenile common toads.

### Statistical analysis

We considered a specific bufadienolide to be present if its signal-to-noise ratio was at least three in the chromatogram (Hettyey et al. 2019; Üveges et al. 2019, 2021). We estimated the quantity of each compound from the area values of MS chromatogram peaks based on the calibration curve of the marinobufotoxin standard. We subsequently summed these values to obtain estimates of the total bufadienolide quantity (TBQ) for each individual. This approach results in rough estimates of bufadienolide content, but because commercially available standards are lacking for most bufadienolide compounds, this is currently the best quantification method available (Benard and Fordyce 2003; Hagman et al. 2009; Üveges et al. 2021).

For data analysis, the CORT release rate for each tadpole was expressed as the total amount of CORT released per hour, divided by tadpole dry mass (pg/mg/h). Toxin content was expressed as mass-corrected TBQ (mcTBQ, ng/mg) by dividing the TBQ by tadpole dry mass. The response variables were adjusted to body mass to account for the potential allometric relationship between body mass and TBQ or CORT release rate (Fig. S1), and because body mass and developmental stage were highly correlated (Fig. S2).

**Fig. 3:**
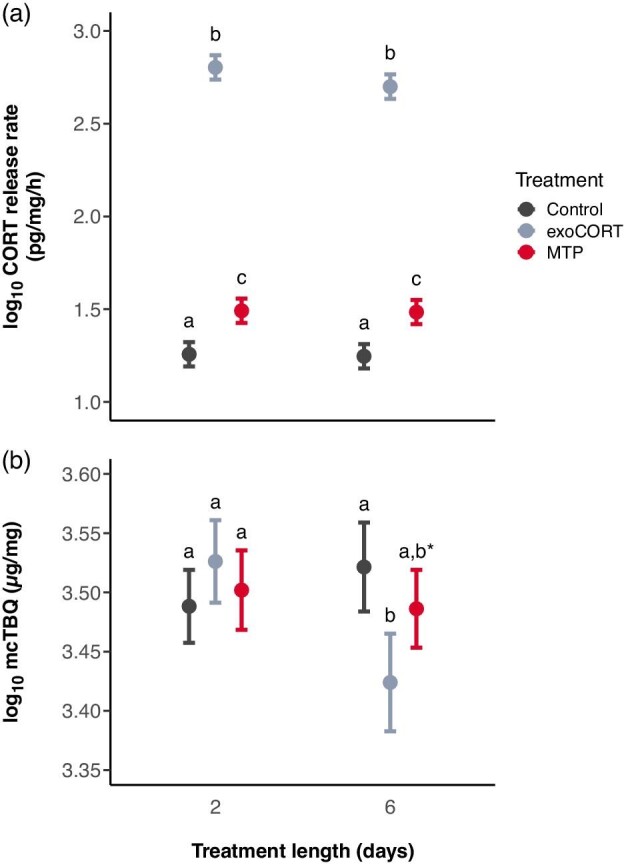
Effects of treatments (exoCORT: exogenous corticosterone, MTP: metyrapone) on corticosterone (CORT) release rate (**a**) and mass-corrected total bufadienolide content (mcTBQ, **b**) of toad tadpoles. Error bars represent estimated means and 84% confidence intervals (CI) calculated from linear mixed-effects models (*N* = 283). Nonoverlapping CIs indicate significant differences between groups after correction for false discovery rate. Letters above the error bars represent pairwise comparisons; groups not sharing any letter differ significantly (*P* < 0.05). An asterisk depicts a marginally non-significant difference (*P* = 0.071) between groups treated with exoCORT and MTP.

All statistical analyses were run in R 4.0 (R Development Core Team 2017) using linear mixed-effects models. We log_10_-transformed both CORT release rate and mcTBQ to meet the model assumptions based on diagnostic residual plots. We used the “lmer” function of the “lme4” package (Bates et al. 2015) to analyze CORT release rate. We included treatment length (2 or 6 days), presence or absence of predation cues, and the type of exogenous chemicals (exoCORT, MTP, or EtOH) as fixed factors, and developmental stage centered to the mean as a covariate, as well as all two-way interactions between the fixed factors and their three-way interaction. We included clutch and block as crossed random factors. To analyze log_10_-transformed bufadienolide content, we used the same model structure as for CORT release rate, except that clutch was used as the sole random factor. Preliminary analysis of data indicated that the block had no effect on mcTBQ, therefore, it was omitted from this model. To account for differences in variances between the twelve treatment combinations and to improve model fit, we included the “weights” argument with the “varIdent” function in the mcTBQ model, which was run with the “lme” function of the “nlme” package (Pinheiro et al. 2017). We obtained *P* values from type-2 analysis-of-deviance (ANOVA) tables implemented in the “car” package (Fox and Weisberg 2019). We conducted post-hoc pairwise comparisons among treatment groups using linear contrasts implemented in the “emmeans” package (Lenth 2023). *P* values were corrected for multiple comparisons by using the false discovery rate (FDR) method (Benjamini and Hochberg 1995; Pike 2011). We present the results of pairwise comparisons using means ± 84% confidence intervals (CI), as suggested by Payton et al. (2003) for assessment of equality among groups (Fig. 3). Nonoverlapping CIs indicate significant differences between groups after correction for FDR. Additionally, we tested Pearson's product–moment correlation implemented in base R and partial correlation controlling for the developmental stage using the “ppcor” package (Kim 2015) between log_10_ transformed CORT release rate and log_10_ transformed mcTBQ.

## Results

The CORT release rate of tadpoles was significantly affected by exogenous chemicals (Table 1). Tadpoles exposed to exoCORT released significantly more CORT compared to the control and MTP-treated groups, while tadpoles in the MTP group exhibited intermediate CORT release rates, differing significantly from those in the control and exoCORT groups (Table 2, Fig. 3a). The effect of developmental stage on the CORT release rate was also significant: more developed tadpoles excreted lower amounts of CORT (Table 1, Fig. S3A). Treatment length, predation cues, and their interactions had no significant effect on CORT release rates (Table 1).

**Table 1:** Treatment effects (type-2 analysis of deviance) on corticosterone (CORT) release rate and mass-corrected bufadienolide quantity (mcTBQ) of toad tadpoles.

Response	Effect	*χ* ^2^	df	*P*
CORT release rate	**Developmental stage**	57.776	1	**<0.001**
	**Exogenous chemicals**	2674.305	2	**<0.001**
	Predation cues	0.456	1	0.499
	Treatment length	2.365	1	0.124
	Exogenous chemicals × predation cues	3.024	2	0.221
	Exogenous chemicals × treatment length	3.079	2	0.215
	Predation cues × treatment length	0.193	1	0.661
	Exogenous chemicals × predation cues × treatment length	0.91	2	0.634
mcTBQ	**Developmental stage**	272.422	1	**<0.001**
	Exogenous chemicals	0.559	2	0.756
	Predation cues	0.014	1	0.906
	Treatment length	1.257	1	0.262
	Exogenous chemicals × predation cues	0.071	2	0.965
	**Exogenous chemicals × treatment length**	10.327	2	**0.006**
	Predation cues × treatment length	2.28	1	0.131
	Exogenous chemicals × predation cues × treatment length	3.44	2	0.179

Significant effects are highlighted in bold.

**Table 2:** Effects of exogenous chemicals on corticosterone (CORT) release rate and mass-corrected total bufadienolide quantity (mcTBQ) of toad tadpoles.

Response	Treatment length (days)	Contrast	Estimate	SE	df	*t*	*P*
CORT release rate	2	**exoCORT—MTP**	–1.312	0.044	261	–30.067	**<0.001**
	2	**EtOH—exoCORT**	1.546	0.044	261	35.423	**<0.001**
	2	**EtOH—MTP**	0.234	0.044	261	5.376	**<0.001**
	6	**exoCORT—MTP**	–1.215	0.044	262	–27.711	**<0.001**
	6	**EtOH—exoCORT**	1.453	0.045	263	32.653	**<0.001**
	6	**EtOH—MTP**	0.238	0.043	262	5.491	**<0.001**
mcTBQ	2	exoCORT–—MTP	–0.024	0.028	264	–0.855	0.590
	2	EtOH—exoCORT	0.038	0.027	264	1.401	0.487
	2	EtOH—MTP	0.014	0.026	264	0.518	0.605
	6	exoCORT—MTP	0.062	0.031	264	1.991	0.071
	6	**EtOH—exoCORT**	–0.097	0.034	264	–2.898	**0.012**
	6	EtOH—MTP	–0.035	0.030	264	–1.194	0.233

Estimates of linear contrasts are differences between the mean values of tadpoles exposed to the solvent control (EtOH), exogenous CORT (exoCORT), or metyrapone (MTP). *P* values were corrected using the FDR method for each dependent variable. Significant differences are highlighted in bold.

mcTBQ of tadpoles was significantly affected by the interaction between treatment length and exposure to exogenous chemicals (Table 1). In the 6-day treatments, tadpoles exposed to exoCORT contained significantly lower mcTBQ than control tadpoles and marginally nonsignificantly lower mcTBQ than tadpoles exposed to MTP, while the toxin content of tadpoles in the control group and those exposed to MTP did not differ significantly from each other (Table 2, Fig. 3b). Conversely, in tadpoles treated for 2 days, mcTBQ did not differ among treatments. The developmental stage of tadpoles significantly influenced mcTBQ, where more developed tadpoles contained lower amounts of bufadienolides per unit mass (Table 1, Fig. S3B). Predator treatment and its interactions had no significant effect on mcTBQ (Table 1).

Mass-corrected bufadienolide quantity significantly and positively correlated with CORT release rate (*r* = 0.226, *P* < 0.001, Fig. S4). However, this relationship was driven by developmental stage (Fig. S3): when we controlled for this covariate the correlation was no longer significant (*r* = 0.049, *P* = 0.414).

## Discussion

Treating tadpoles with exogenous CORT resulted in significantly increased CORT release rates, regardless of treatment length, and this effect was accompanied by a decrease in bufadienolide toxin content after 6 days. Based on these results, we can reject the hypothesis that the chemical defense of toad tadpoles is upregulated by increased CORT levels (H1, Fig. 1). The observed relationship between CORT release rate and bufadienolide content in our study is in concordance with previous findings indicating that exposure to the amphibian chytrid fungus (*Batrachochytrium dendrobatidis* [Bd]), an important amphibian pathogen, induces increased CORT levels in multiple amphibian species (e.g., Gabor et al. 2013b, 2015, 2018, but see Hammond et al. 2020), but decreases bufadienolide content of common toad tadpoles (Ujszegi et al. 2021; Kásler et al. 2022). We caution, however, against interpreting our result as CORT inhibiting bufadienolide synthesis, for the following reasons. First, multiple studies report that various environmental stressors (e.g., intraspecific competitors, predators, anthropogenic pollutants) known to increase CORT levels in animals (e.g., Glennemeier and Denver 2002a; Berger et al. 2007; Eraud et al. 2008; Middlemis Maher et al. 2013; Braasch et al. 2014; Ye et al. 2015; Gabor et al. 2018; Monclús et al. 2018) also lead to increased bufadienolide production in toads (Bókony et al. 2017, 2018; Hettyey et al. 2019; Üveges et al. 2021). Second, we found that individual variation in bufadienolide content was not correlated with variation in CORT release rate after controlling for the developmental stage. We propose instead that our results on the effects of exoCORT treatment indirectly support the second hypothesis of our study (H2, Fig. 1), that is, that bufadienolide synthesis is induced by upstream hormones of the HPI axis, such as ACTH (Fedorova et al. 1998; Dostanic-Larson et al. 2005). Although we did not measure ACTH in our experimental tadpoles, treating them with exoCORT most probably led to the suppression of ACTH discharge due to negative feedback (Gjerstad et al. 2018; Godoy et al. 2018; Herman et al. 2020), which may have ultimately resulted in decreased bufadienolide content. While our study cannot provide direct evidence for the role of upstream brain hormones in regulating bufadienolide production in toads (H2, Fig. 1), this remains a probable pathway, since previous studies suggested ACTH-induced synthesis of endogenous cardiotonic steroids (Vinge et al. 1993; Laredo et al. 1994, 1995; Li et al. 1994; Yamada et al. 1997; Sophocleous et al. 2003; Dostanic-Larson et al. 2005), including unidentified bufadienolide-like compounds (Fedorova et al. 1998), in mammals.

We expected that treatment with MTP would lead to decreased CORT release rates compared to control animals, as in previous studies on amphibians (Glennemeier and Denver 2002a, 2002b). However, we found instead that MTP slightly, albeit significantly, increased CORT release rates compared to control tadpoles, regardless of treatment length. Such inconsistent effects of MTP treatment have been reported previously (Glennemeier and Denver 2002a; Kulkarni et al. 2017; Gabor et al. 2018; Kennedy et al. 2020). There may be multiple, mutually nonexclusive explanations for the inability of MTP to reduce CORT. First, it is likely that the MTP concentration used in our study did not entirely impede CORT synthesis (Glennemeier and Denver 2002a, 2002b). Therefore, the elevated level of CORT observed in tadpoles treated with MTP may have simply been a consequence of compensatory ACTH release due to decreased CORT synthesis (Coppage et al. 1959; McEwen 2007). Second, MTP inhibits aldosterone (ALDO) synthesis by inhibiting the transformation of 11-deoxycorticosterone to CORT and then that of CORT to ALDO (Gower 1974). ALDO is a potent mineralocorticoid, regulating natriuresis and water retention (Liddle 1958), and decreased levels of this hormone lead, among others, to increased urine volume (Liddle 1958; Brismar et al. 1985; Costello-Boerrigter et al. 2003). Since in our study we measured CORT release rate of tadpoles, it is possible that the apparently increased CORT in response to MTP is a consequence of increased urine (and this way CORT) excretion due to the inhibition of ALDO synthesis.

We observed a decrease in bufadienolide content only after a 6-day exposure period to exoCORT. It is well known that systemic effects of GCs fundamentally differ when high levels are sustained only acutely *versus* chronically (Sterling and Eyer 1988; McEwen 1998, 2004; McEwen and Wingfield 2003; Denver 2009; Middlemis Maher et al. 2013; Godoy et al. 2018). Effects of acute exoCORT exposure, or spikes in innate CORT levels, are relatively quickly counteracted by other physiological processes, and the concentrations of CORT and upstream hormones return to baseline levels (McEwen 1998; McEwen and Wingfield 2003; Romero et al. 2009; McEwen et al. 2015). On the other hand, chronically high levels of CORT can induce emergency countermeasures or even pathological changes in a multitude of physiological processes (Sterling and Eyer 1988; McEwen 1998, 2004; McEwen and Wingfield 2003; Denver 2009; Godoy et al. 2018). Such effects of the 6-day exoCORT treatment might have led to a decrease in bufadienolide content by, for example, the breakdown of the acidic bile-acid pathway of steroidogenesis (Fedorova et al. 2015), or depletion of toxin reserves due to catabolism of toxin compounds for energy release.

Lastly, exposure to predation cues did not result in increased CORT release rates in experimental tadpoles. It is possible that cues on the presence of predators are relatively weak triggers of the endocrine stress response in common toad tadpoles. For instance, induced responses against predators in, for example, behavior and morphology are accompanied by increased CORT levels in amphibians (Middlemis Maher et al. 2013; Kulkarni and Gramapurohit 2017), but common toad tadpoles display less intense behavioral and morphological plasticity to the presence of predators compared to larvae of other amphibians (Laurila et al. 1998; Lardner 2000; Van Buskirk 2002). This is possibly due to the effectiveness of bufadienolides providing defense against various predator species even at non-induced levels (Üveges et al. 2019). Future studies similar to the current experiment should use a higher concentration of predator cues or other types of (possibly more effective) stressors to trigger the upregulation of CORT release rates in toad tadpoles.

Bufadienolides are emerging as important compounds for multiple fields of research. They are considered to be important ion-transport regulators and agents of cell signaling (Pierre and Xie 2006; Schoner and Scheiner-Bobis 2007; Lingrel 2010; Lichtstein et al. 2012); however, to the best of our knowledge, studies investigating the housekeeping aspect of bufadienolides are entirely lacking outside of mammals. Therefore, it would be worthwhile for future studies to also focus on nonmodel vertebrates. Investigating how bufadienolide production influences other physiological processes would also be welcome. For instance, bufadienolides can also inhibit steroidogenesis (Kau et al. 2012); therefore, changes in bufadienolide levels may also influence sex-hormone synthesis. The consequences of this interaction are currently not known, but could potentially impact individual fitness and thus population dynamics and survival in the long run (Bókony et al. 2019). A deeper understanding of the regulation of these compounds might also benefit native populations suffering from invasive toad species (Shine 2010; Licata et al. 2019), by, for example, potentially leading to methods for selective inhibition of bufadienolide synthesis, thereby facilitating the survival of toad predators (Phillips and Shine 2006; Greenlees et al. 2010; Caller and Brown 2013). Last but not least, research on how these housekeeping molecules became effective chemical weapons may also provide important insights into molecular evolution. Clearly, more research is needed to shed light on the background of phenotypically plastic bufadienolide synthesis, and its potential effects on the physiology of animals, as well as its ecological and evolutionary consequences.

## Supplementary Material

obad021_Supplemental_File

## Data Availability

Dataset of the study is available on the figshare digital repository at https://doi.org/10.6084/m9.figshare.15090339 (Üveges et al. 2023).
