# Supplementary material for: Does the Glucocorticoid Stress Response Make Toads More Toxic? An Experimental Study on the Regulation of Bufadienolide Toxin Synthesis
Source: Integr Org Biol. 2023 Jun 5;5(1):obad021. doi: 10.1093/iob/obad021 (PMC10331804; doi:10.1093/iob/obad021)
Supplement: obad021_Supplemental_File [file obad021_supplemental_file.docx]

**Supplementary material**

**Supplementary methods***Detailed methods of toxin sample preparation and bufadienolide analysis*
We prepared samples by homogenising preserved tadpoles using an IKA S25N-8G dispersing tool attached to an IKA Ultra Turrax-T 25 homogenizer. Subsequently, we dried homogenates *in vacuo* at 45 °C using a Büchi Rotavapor R-134 rotary evaporator and measured dry mass to the nearest 0.1 mg with an analytical balance (Sartorius Entris 224i-1S). Samples were re‐dissolved in 1 ml HPLC‐grade absolute methanol, facilitated by brief exposure to ultrasound in a bath sonicator (Tesla UC005AJ1). Finally, we filtered samples using FilterBio nylon syringe filters (pore size = 0.22 μm).

We analysed samples using high-performance liquid chromatography with diode-array detection and mass spectrometry (HPLC-DAD-MS). We co-injected the following bufadienolides as standards: bufalin, bufotalin, resibufogenin, gamabufotalin, areno- and telocinobufagin (Biopurify Phytochemicals, Chengdu, China), cinobufagin (Chembest, Shanghai, China), cinobufotalin (Quality Phytochemicals, New Jersey, USA), digitoxigenin (Santa Cruz Biotechnology, Dallas, TX, USA) and marinobufotoxin (courtesy of Prof. Rob Capon, University of Queensland, Brisbane, Australia). To help identify bufadienolide compounds present in low quantities, we also analysed a bulk sample from 49 juvenile common toads obtained by manually applying pressure to their parotoid glands.

We quantified bufadienolide compounds using a single-quadrupole HPLC-MS system (Model LC-MS-2020, Shimadzu, Kyoto, Japan) equipped with a binary gradient solvent pump, a vacuum degasser, a thermostated autosampler, a column oven, a photodiode detector and a mass analyser with electrospray ionization (ESI/MS). Ten µl of samples were injected at 35 °C on a Kinetex C18 2.6 µm column (100 mm x 3 mm i.d.) in series with an octadecyl C18 guard column (4 mm × 3 mm i.d.). Eluent A was 5 % aqueous acetonitrile with 0.05 % formic acid, eluent B was acetonitrile with 0.05 % formic acid. The flow rate was 0.6 ml/min and the gradient was as follows: 0-1 min: 10-20 % B; 1-11 min: 20-29 % B; 11-13 min: 29-58 % B; 13.1-16 min: 100 % B; 16.1-20 min: 10 % B. ESI conditions were set as follows: interface temperature: 350 °C; desolvation line (DL) temperature: 250 °C; heat block temperature: 400 °C; drying N_2_ gas flow: 15 L×min-1; nebulizer N_2_ gas flow: 1.5 L×min-1; positive ionization mode. Full scan spectra were recorded in the range of 350–800 m/z and we also performed selected-ion monitoring (SIM) detecting the base peaks of bufadienolides we previously found in common toads (Üveges et al., 2017; Bókony et al., 2018; Hettyey et al., 2019). Data were processed using the LabSolutions 5.42v software (Shimadzu, Kyoto, Japan).

**Fig. S1:** Relationship between body mass and corticosterone (CORT) release rate (**A**) or total bufadienolide quantity (TBQ, **B**) of toad tadpoles. Abbreviations: exoCORT: exogenous corticosterone, MTP: metyrapone.


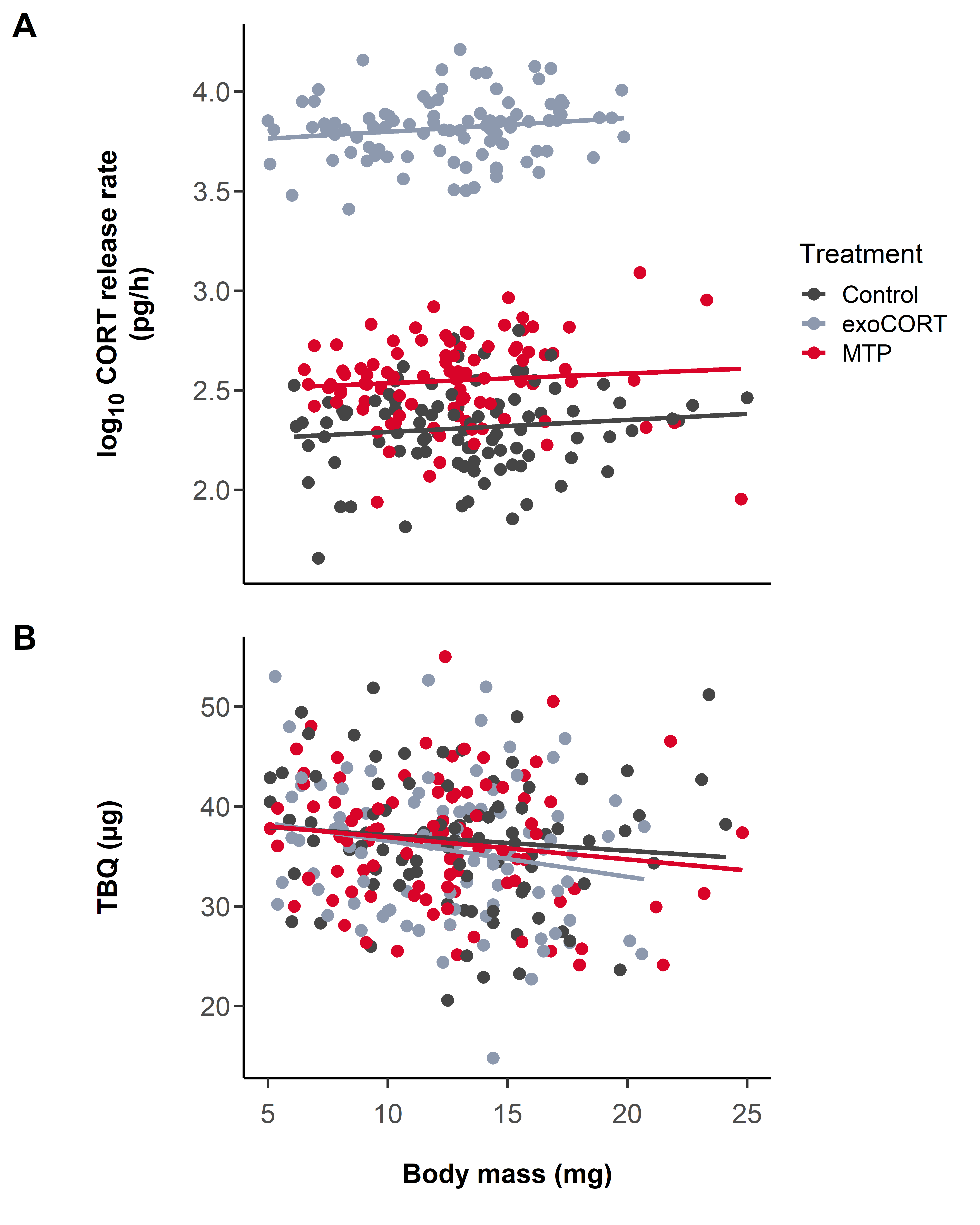


**Fig. S2:** Relationship between body mass and developmental stage of toad tadpoles. Abbreviations: exoCORT: exogenous corticosterone, MTP: metyrapone.


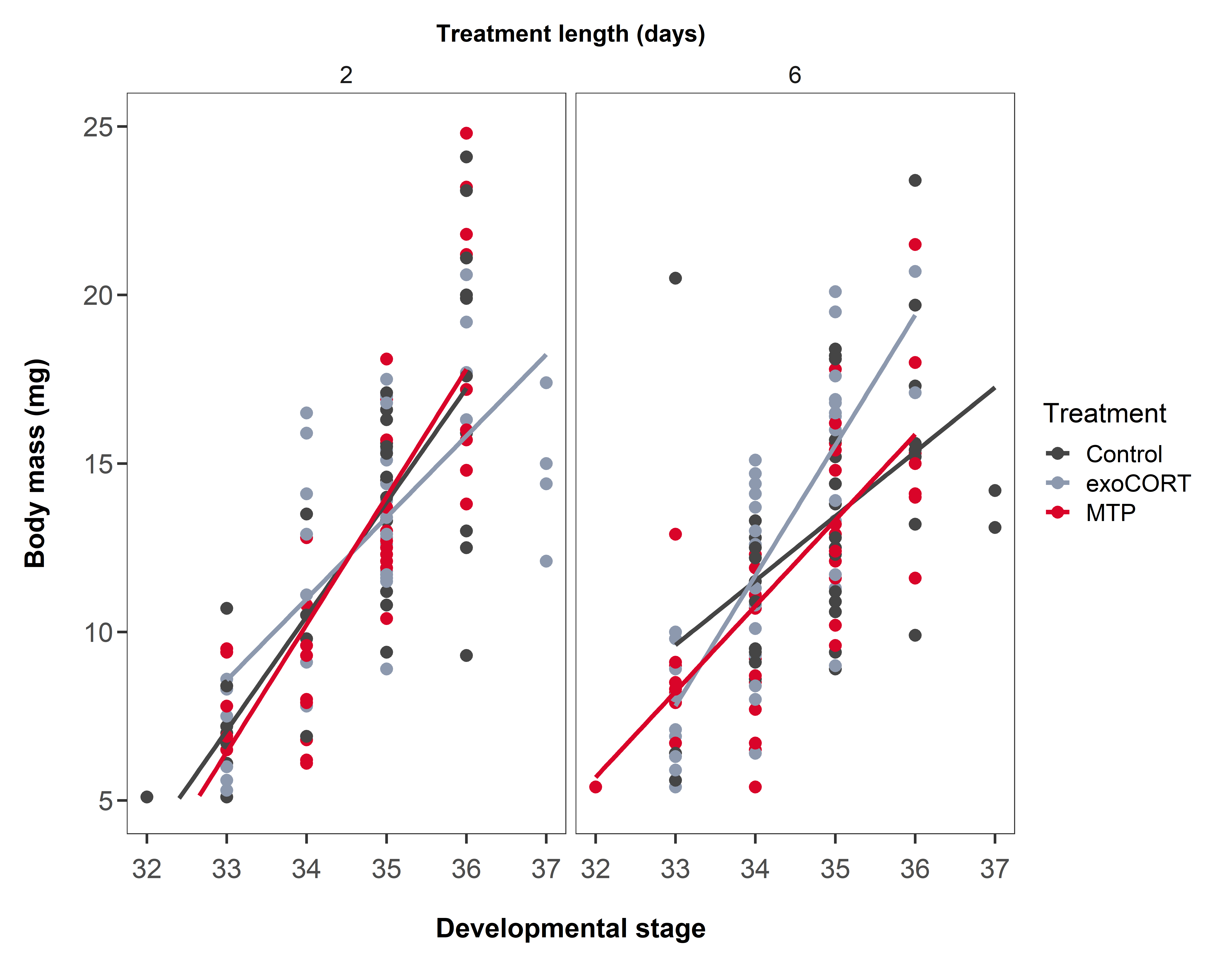


**Fig. S3:** Relationship between developmental stage and corticosterone (CORT) release rate (**A**) or mass-corrected total bufadienolide quantity (mcTBQ, **B**) of toad tadpoles. Abbreviations: exoCORT: exogenous corticosterone, MTP: metyrapone.


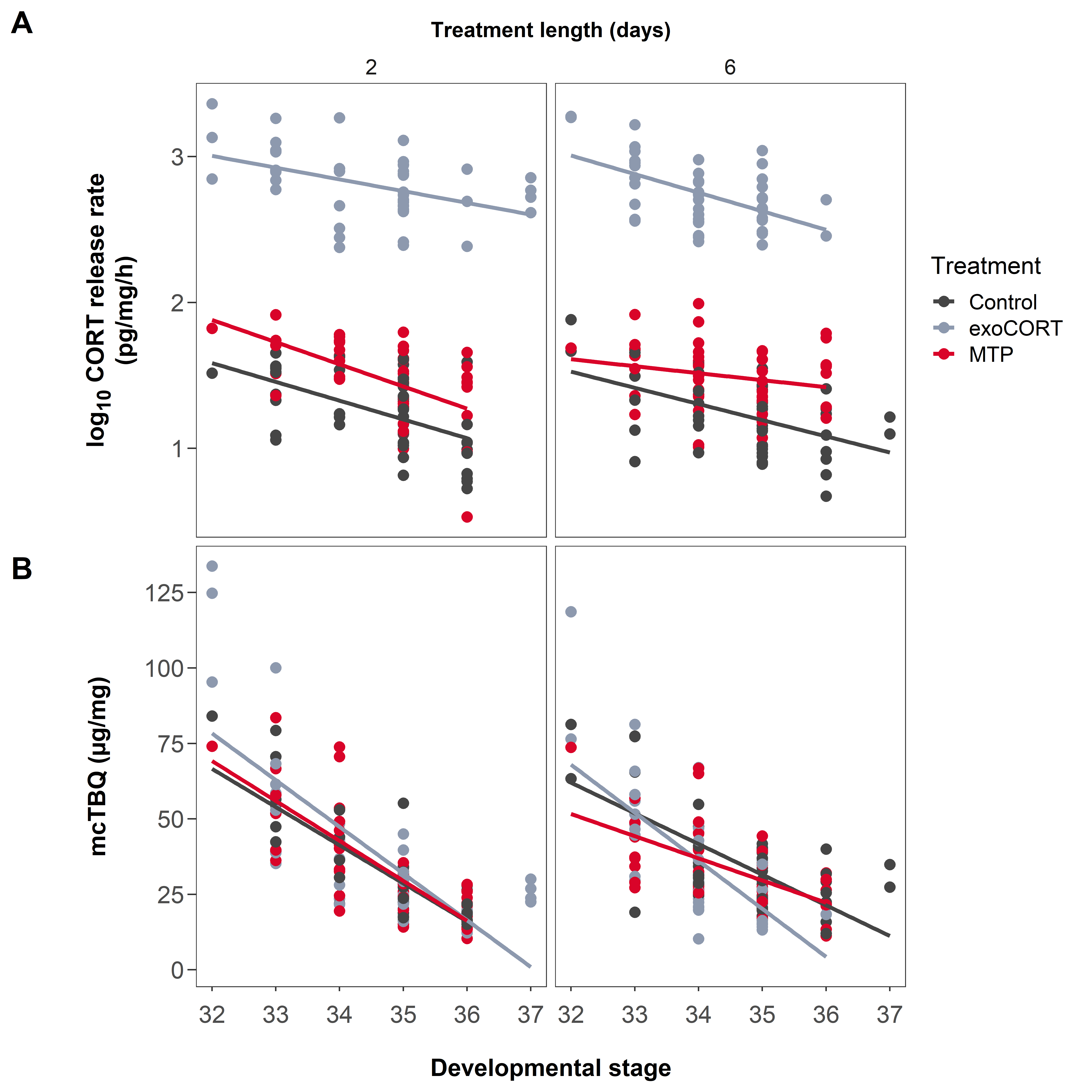


**Fig. S4:** Relationship between corticosterone (CORT) release rate and mass-corrected total bufadienolide quantity (mcTBQ) of toad tadpoles. Abbreviations: exoCORT: exogenous corticosterone, MTP: metyrapone.

**
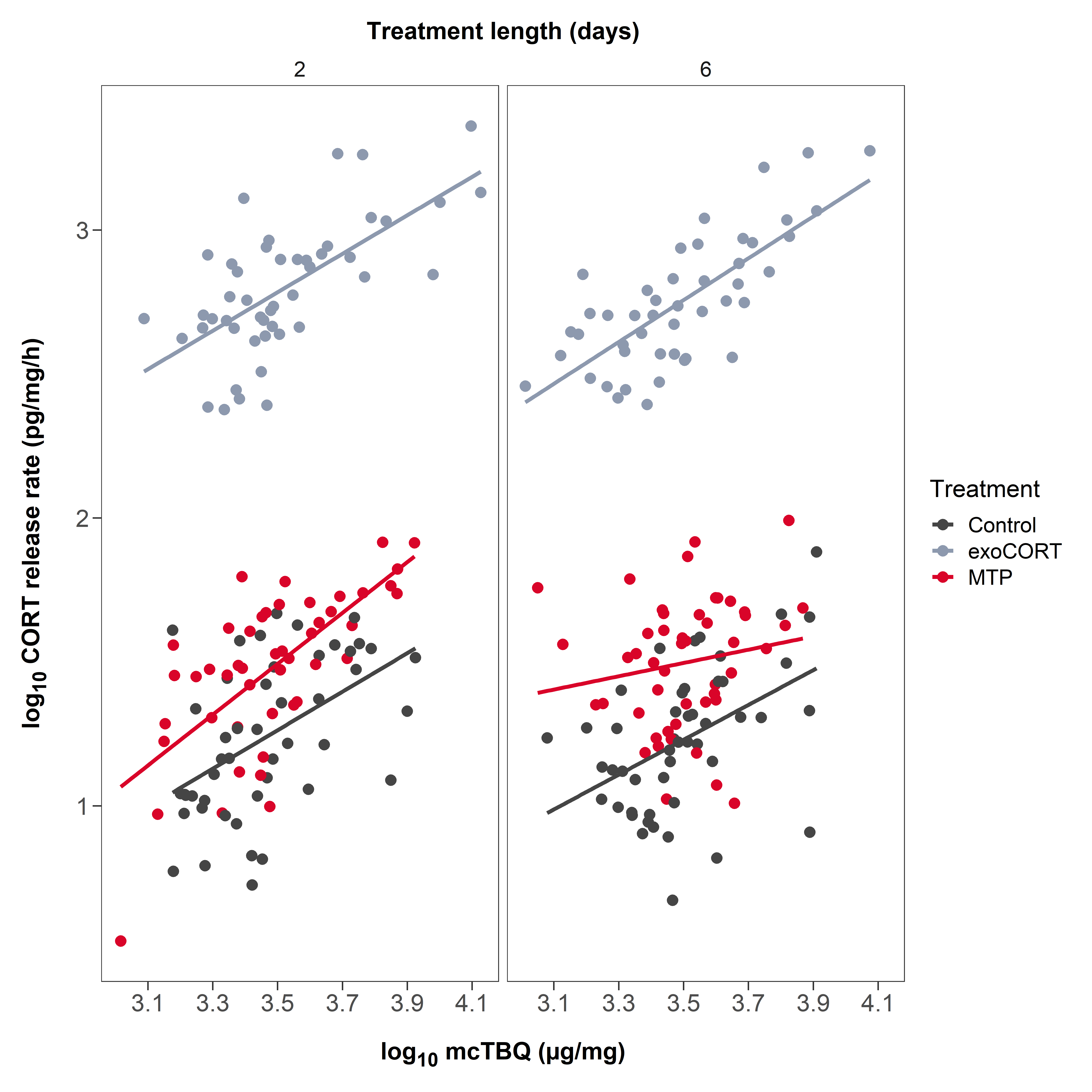
**
